# Hepatitis C virus seroprevalence and prevalence of chronic infection in the adult population in Ireland: a study of residual sera, April 2014 to February 2016

**DOI:** 10.2807/1560-7917.ES.2017.22.30.30579

**Published:** 2017-07-27

**Authors:** Patricia Garvey, Brian O'Grady, Geraldine Franzoni, Maeve Bolger, Katie Irwin Crosby, Jeff Connell, Deirdre Burke, Cillian De Gascun, Lelia Thornton

**Affiliations:** 1 Health Service Executive - Health Protection Surveillance Centre, Dublin, Ireland; 2 European Programme for Intervention Epidemiology Training (EPIET), European Centre for Disease Prevention and Control (ECDC), Stockholm, Sweden; 3 National Virus Reference Laboratory, University College Dublin, Dublin, Ireland

**Keywords:** Seroepidemiologic studies, hepatitis C chronic, hepatitis C, cross-sectional studies

## Abstract

Robust data on hepatitis C virus (HCV) population prevalence are essential to inform national HCV services. In 2016, we undertook a survey to estimate HCV prevalence among the adult population in Ireland. We used anonymised residual sera available at the National Virus Reference Laboratory. We selected a random sample comprising persons ≥ 18 years with probability proportional to the general population age-sex distribution. Anti-HCV and HCV Ag were determined using the Architect anti-HCV and HCV Ag assays. Fifty-three of 3,795 specimens were seropositive (age-sex-area weighted seroprevalence 0.98% (95% confidence interval (CI): 0.73–1.3%)). Thirty-three specimens were HCV-antigen and antibody-positive (age-sex-area weighted prevalence of chronic infection 0.57% (95% CI: 0.40–0.81%)). The prevalence of chronic infection was higher in men (0.91%; 95% CI: 0.61–1.4%), in specimens from the east of the country (1.4%; 95%CI: 0.99–2.0%), and among persons aged 30–39 years and 40–49 years (1.1% (95% CI: 0.59–2.0%) and 1.1% (95% CI: 0.64–1.9%) respectively). Ireland ranks at the lower end of the spectrum of prevalence of chronic HCV infection internationally. Men born between 1965 and 1984 from the east of the country have the highest rate of chronic HCV infection.

## Background

Acute hepatitis C virus (HCV) infection is typically asymptomatic or associated with non-specific symptoms. Studies have indicated, however, that up to 80% of those infected will develop chronic infection, which can lead, over many decades, to cirrhosis, liver cancer and death [1,2]. Because of the asymptomatic nature of HCV infection, individuals can be infected for many years before diagnosis. Globally, the World Health Organization (WHO) has estimated that between 130 and 150 million people are HCV-infected [3,4], with the prevalence of HCV in some countries in central Asia (5.4%), western Africa (5.3%), central Africa (4.2%), eastern Europe (3.3%), and North Africa/Middle East (3.1%) being higher than countries in North America (1.0%) and western Europe (0.9%) [5]. Within Europe, prevalence estimates of 0.4% to 5.2% have been reported, with countries in the north and west of Europe having lower estimates (0.9%) than countries in the east of Europe (3.3%) [5,6].

Historically, there was limited success in treating HCV, but in recent years, treatment with new direct-acting antivirals (DAAs) that possess high efficacy and improved safety profiles, has led many to suggest that the elimination of HCV is now possible [7]. Successful treatment not only benefits the individual by reducing his or her risk of cirrhosis and other liver-related outcomes, but also benefits the general population by reducing rates of onward transmission.

With the advent of highly potent and curative DAAs, many countries are now developing national strategies for population screening for HCV infection, and national HCV treatment programmes. Initiatives in Ireland include the formal establishment in 2015 by the Health Service Executive (HSE) of a National Hepatitis C Treatment Programme for known HCV-infected individuals [8]. Concurrently, a Guideline Development Group was convened by the HSE to develop national HCV screening guidelines to identify HCV-infected individuals who are currently unaware of their HCV status. For these approaches to be successful, the availability of robust data on population HCV seroprevalence is key, a fact recognised both by the Irish National Hepatitis C Strategy 2011–2014 [9], and likewise in December 2015, by the European Centre for Disease Prevention and Control (ECDC) [10].

Ireland is believed to be a low-prevalence country for HCV, and prior studies that measured the HCV seroprevalence in selected high-risk or localised populations, and in antenatal women [11-16], support this view; however, no national HCV prevalence studies in the general population have been conducted and the true burden of infection is unknown. We undertook a national cross-sectional study to estimate HCV seroprevalence and prevalence of HCV chronic infection among the adult population in Ireland.

## Methods

### Study design and population

The target population for our study was the adult population in Ireland. The sample was based on anonymised residual sera taken from persons aged 18 years or over submitted to the National Virus Reference Laboratory (NVRL). The NVRL provides a diagnostic and reference service for clinicians investigating viral infections throughout Ireland. Typically, around 200,000 blood specimens are received annually, equating to ca 150,000 serum specimens. They include specimens received for diagnostic purposes, antenatal screening, and pre-employment screening.

### Laboratory residual sera

Specimens are classified as residual at the point where they are deemed no longer required for the purpose for which they were originally collected. It is NVRL policy to retain diagnostic samples for 4 months, antenatal samples for 24 months, needlestick source samples for 24 months, and occupational health screening specimens as requested. These time periods are intended to facilitate supplementary testing of the original sample should a clinical need arise. After the relevant time period has elapsed however, samples are discarded. For this study, residual specimens were available at the NVRL for a 3-month period for those specimens normally retained for 2 years (specimens collected April to June 2014), and for a 4-month period for those specimens normally retained for a 4-month period (specimens collected November 2015 to February 2016). Laboratory testing for the purposes of this study took place in July and August 2016.

### Sample size

We estimated a sample size of 3,814 corresponding to an expected prevalence of chronic infection of 0.5%, an absolute precision of 0.2%, an alpha error of 0.05, a design effect of 1, and an eligible pool of ca 18,891 specimens.

### Sampling procedure

The NVRL laboratory information management system was used to identify eligible specimens. Specimens marked on the system as being specifically retained for other reasons, e.g. sample from organ donors, were excluded. Antenatal specimens (where submitted for general antenatal screen at first ‘booking’) and pre-employment screening specimens were all included in the sampling frame. To avoid the over-representation of persons who could have a HCV prevalence higher than expected in the general population, specimens collected from certain sources were considered ineligible for the study. These included: specimens sourced from drug treatment clinics or sexually transmitted infection (STI) clinics; specimens from hepatology or infectious disease services; specimens submitted specifically for a hepatitis, or STI screen; or specimens from asylum seekers (who are routinely screened for HCV in Ireland). Where possible, duplicate specimens from the same individual were identified by individually cross-checking the submission details for specimens from patients with the same initials and date of birth, and only one specimen per person was included in the sampling frame. We stratified the eligible specimens in the sampling frame by age group and sex. We selected a sample with probability proportional to the size of the age group and sex strata in the general population (as specimens submitted for diagnostic tests are likely to be biased at least by age). Within age-sex strata, we selected specimens using simple random sampling.

### Laboratory specimen analysis

Where a selected specimen was found to have insufficient volume (<500 µL) for conducting the required laboratory tests, a replacement was selected randomly from the remaining specimens in the sample frame.

HCV antibody status was determined using the HCV Ab Architect Abbott HCV antibody test (Abbott Diagnostics, Wiesbaden, Germany), which detects only anti-HCV IgG, as the first line screen. Specimens exceeding the manufacturer’s cut-off of 1.0 were investigated for the presence of HCV antigen using the HCV Ag Architect Abbott HCV antigen test. Samples reactive in the anti-HCV assay but negative for HCV Ag were subsequently tested using the Bio-Rad Monolisa anti-HCV Plus vs 3.0 (Bio-Rad, Marnes-la-Coquette, France) to confirm the presence of anti-HCV. Specimens which generated discordant anti-HCV results were tested using the Fujirebio INNO-LIA HCV score line immunoassay (Fujirebio Europe, Gent, Belgium) to determine the anti-HCV status of the sample.

### Definitions

Specimens that were both HCV-antigen- and antibody-positive were considered to have been collected from an individual with chronic HCV infection. Specimens that were anti-HCV-positive but HCV-antigen-negative were considered indicative of resolved infection. Specimens that were HCV-antigen-positive but that gave an indeterminate anti-HCV profile were considered as having been obtained from a subject with possible acute HCV infection. Specimens with indeterminate anti-HCV antibody status and that were HCV-antigen-negative were recorded as being of inconclusive HCV status.

### Information collected

As the specimens used were derived from residual sera that had been submitted for a wide variety of reasons, the only information common to all specimens comprised basic demographic data such as age, sex, geographic area (Health Service Executive (HSE) areas are the public health administrative units in Ireland) and sample category (pre-employment screening, antenatal or other). These data were linked with the laboratory results in the study database.

### Statistical analysis

We calculated the prevalence of HCV antibodies and chronic HCV infection and 95% confidence intervals (overall and by age, sex and area), weighted for under-sampling in some age-sex strata and for geographical bias in sample selection. As a comparison group for antenatal studies previously conducted in Ireland, we also calculated the weighted prevalence of HCV antibodies in specimens from women aged 18–49 years.

Extrapolating from the prevalence in the sample, we estimated the number of persons seropositive or chronically HCV infected in the adult population in Ireland. Stata version 14.0 (Stata Corporation, Texas, US) statistical software was used for analyses

### Protection of human subjects and confidentiality

The study received ethical approval from the Royal College of Physicians of Ireland (RCPI) Research Ethics Committee. All testing was anonymous and the identities of those whose specimens were tested were unknown to investigators. No contact was made with these individuals and they were unaware that they were included in the study. Before testing, eligible specimens were decanted and irrevocably anonymised using new specimen numbers. Only these new anonymised specimen numbers were recorded in the database.

## Results

### Construction of the study sampling frame

NVRL database records for serological specimens received during the period of interest (due for discard March to June 2016) were reviewed to see if they met the study inclusion and exclusion criteria. Excluding records for specimens flagged for specific retention for other reasons, and those from persons less than 18 years of age, records were available for 49,269 specimens.

**Figure 1:**
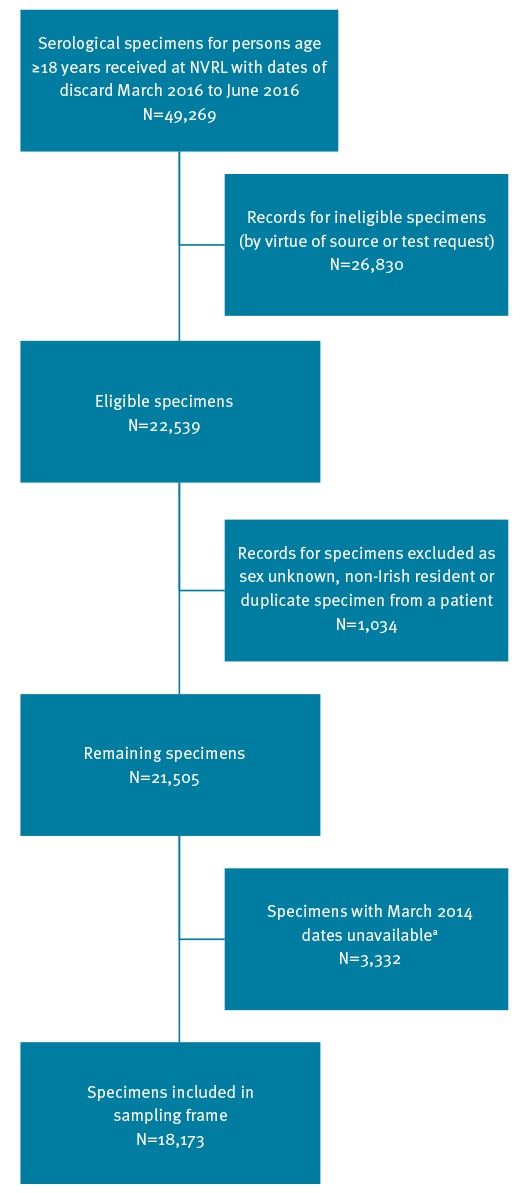
Construction of hepatitis C virus study sampling frame, Ireland, 2014–2016

10,382 records were identified for antenatal screening specimens and 615 records for pre-employment screen specimens. After exclusions were applied to the 38,372 records for diagnostic specimens, 11,542 records remained, which, when combined with the antenatal and pre-employment screen records, totalled 22,539 records (Figure 1).

NVRL: National Virus Reference Laboratory

^a^ Specimens collected in March 2014 and due for discard in March 2016 had been inadvertently discarded.

A further 1,034 records were excluded for the following reasons: no sex was recorded, the patient had a non-Irish residential address, or more than one specimen had come from the same individual. At this point, we also became aware that specimens collected in March 2014 and due for discard in March 2016 had already been discarded, leaving 18,173 specimens in the final sampling frame (Figure 1).

Antenatal specimens made up 7,600 (42%), pre-employment screen specimens 499 (3%) and the remaining diagnostic specimens 10,074 (55%) of this sampling frame.

### Demographic and specimen characteristics of study sample

From a sampling frame of 18,173 specimens, a study sample of 3,814 specimens was selected. After including replacements for specimens of insufficient volume (where possible), the final sample comprised 3,795 specimens (99.5% of the desired number), with minimal under-sampling in three age-sex strata. Reflecting the referral bias we might expect in a national service based in the east of the country, the study sample contained a higher proportion of specimens from individuals resident in HSE-East (60%), which includes the greater Dublin area, compared with the general adult population in Ireland (36%) (Table 1). This geographical bias was accounted for in the weighted analysis presented below.

**Table 1:** Demographic characteristics of the study sample (n=3,795) and the general adult population in Ireland (n=3,439,565), 2014–2016

Characteristic		Study sample		General adult population in Ireland (> = 18 years)^a^	
		n	%	n	%
Sex	Female	1,931	51%	1,754,648	51%
	Male	1,864	49%	1,684,917	49%
Age group	18–29 years	856	23%	772,275	22%
	30–39 years	838	22%	758,206	22%
	40–49 years	705	19%	635,997	18%
	50–59 years	574	15%	518,908	15%
	60–69 years	436	11%	392,424	11%
	70 + years	386	10%	361,755	11%
HSE area	East	2,288	60%	1,236,870	36%
	Midlands and North East	535	14%	522,465	15%
	South and South East	556	15%	869,316	25%
	West, North West and Mid West	416	11%	810,914	24%
Category	Antenatal	646	17%	NA	NA
	Pre-employment	131	3%	NA	NA
	Other (e.g. diagnostic specimens)	3,018	80%	NA	NA
Total		3,795	100%	3,439,565	100%

^a^ Census 2011, Central Statistics Office, Ireland

HSE: Health Service Executive; NA: not available.

### Laboratory findings and interpretation

Laboratory findings were consistent with 33 specimens having been collected from patients with chronic HCV infection, 20 from patients with resolved HCV infection, and one from a patient with possible acute infection; 3,737 specimens tested negative for anti-HCV. Four specimens yielded inconclusive anti-HCV results: in the ordinary course of events, the NVRL laboratory testing algorithms would have indicated that further specimens should be requested from these individuals, but this was not possible in this situation. Thus, in this study sample, the HCV chronicity rate was calculated as 62% (33 chronic out of 53 chronic plus resolved infections).

### Seroprevalence

Overall, the 53 specimens confirmed as seropositive (Table 2) corresponded to a weighted seroprevalence of 0.98% (95% CI: 0.73–1.3%). Based on these findings, we estimate that 33,708 people in the adult population in Ireland have had previous exposure to HCV.

**Table 2:** Estimated hepatitis C virus (HCV) seroprevalence and prevalence of chronic HCV infection, and estimated number HCV seropositive and chronically infected, in the adult population in Ireland, by age and sex and Health Service Executive-area, Ireland, 2014–2016

Group		Seropositive in study sample (chronic and resolved infections)			Seropositive adults in general population		Chronically infected adults in study sample			Chronically infected adults in general population	
		Number	Weighted prevalence (%)	95% CI	Number	95% CI	Number	Weighted prevalence (%)	95% CI	Number	95% CI
Sex	Female	14	0.42	0.25–0.71	7,370	4,387–12,458	8	0.24	0.12–0.49	4211	2,106–8,598
	Male	39	1.57	1.12–2.19	26,453	18,871–36,900	25	0.91	0.61–1.37	15333	10,278–23,083
Age	18–29 years	1	0.07	0.01–0.47	541	77–3,630	0	0	0	0	0
	30–39 years	20	1.94	1.21–3.10	14,709	9,174–23,504	12	1.07	0.59–1.95	8113	4,473–14,785
	40–49 years	18	1.53	0.96–2.43	9,731	6,106–15,455	13	1.11	0.64–1.91	7060	4,070–12,148
	50–59 years	6	0.83	0.33–2.09	4,307	1,712–10,845	3	0.30	0.10–0.94	1557	519–4,878
	60–69 years	5	0.69	0.29–1.66	2,708	1,138–6,514	2	0.27	0.07–1.09	1060	275–4,277
	70 + years	3	0.50	0.16–1.57	1,809	579–5,680	3	0.50	0.16–1.57	1809	579–5,680
Area	HSE E	47	2.13	1.60–2.83	26,345	19,790–35,003	31	1.41	0.99–2.01	17440	12,245–24,861
	HSE M+NE	2	0.31	0.08–1.22	1,620	418–6,374	1	0.15	0.02–1.08	784	104–5,643
	HSE S + SE	3	0.48	0.15–1.47	4,173	1,304–12,779	1	0.16	0.02–1.12	1391	174–9,736
	HSE W + NW + MW	1	0.21	0.03–1.45	1,703	243–11,758	0	0	0	0	0
**All**	**Population 18 + years**	**53**	**0.98**	**0.73–1.31**	**33,708**	**25,109–45,058**	**33**	**0.57**	**0.40–0.81**	**19,606**	**13,758–27,860**

CI: confidence interval; E: East; HCV: hepatitis C virus; HSE: Health Service Executive; M: Midlands; MW: Mid West; NE: North East; NW: North West; S: South; SE: South East; W: West.

**Figure 2:**
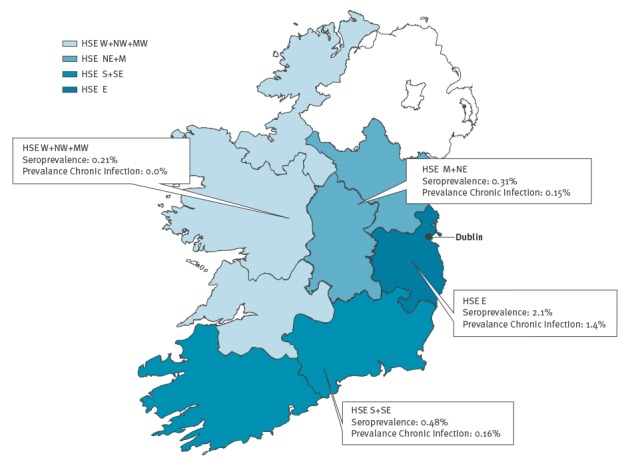
Seroprevalence and prevalence of chronic hepatitis C virus infection by region, Ireland, 2014–2016

HSE: Health Service Executive; M: Midlands; MW: Mid West; NE: North East; NW: North West; S: South; SE: South East; W: West.

Seroprevalence was significantly higher in men (1.6%; 95% CI: 1.1–2.2%) than in women (0.42%; 95% CI: 0.25–0.71%), and in specimens from HSE-East (2.1%; 95% CI: 1.6–2.8%) than in specimens from other areas (Table 2 and Figure 2). Although not statistically significant, there was also a higher seroprevalence among specimens from people aged 30–39 years (1.9%; 95% CI: 1.2–3.1%) and 40–49 years (1.5%; 95% CI: 0.96–2.4%) than in other age groups.

We calculated the weighted seroprevalence among women aged between 18 and 49 years old to be 0.40% (95% CI: 0.20–0.76%).

### Prevalence of chronic infection

The 33 specimens with serology consistent with chronic HCV infection corresponded to a weighted prevalence of chronic infection of 0.57% (95% CI: 0.40–0.81%). Based on this, we estimate that 19,606 persons in the adult population in Ireland have chronic HCV infection (Table 2).

The prevalence of chronic HCV infection was again significantly higher in men (0.91%; 95% CI: 0.61–1.4%) than in women (0.24%; 95% CI: 0.12–0.49%), and higher but not significantly so in specimens from HSE-East (1.4%; 95% CI: 0.99–2.0%) compared with specimens from other areas (Table 2 and Figure 2). There was also a higher prevalence of chronic infection among persons aged 30–39 years and 40–49 years (Table 2), although again, this was not statistically significant. No chronic infections were noted in the 18–29 years age group for either sex, and overall, the highest prevalence of chronic infection was in men aged 40–49 years in HSE-East (5.2%; 95% CI: 2.8–9.3%) and in men aged 30–39 years in HSE-East (3.5%; 95% CI: 1.8–6.9%).

## Discussion

This is the first HCV population prevalence study to have been undertaken in Ireland. Compared with published studies, the estimated prevalence of 0.57% for chronic HCV infection suggests that Ireland ranks at the lower end of the spectrum in terms of HCV prevalence internationally [5,6]. Our findings furthermore suggest that based on age, sex, and geographical area, men born between 1965 and 1984 from the east of the country have the highest rate of chronic HCV infection in Ireland.

Our findings are broadly in line with those of a previous study that calculated a chronic HCV infection rate based on the number of new HCV laboratory diagnoses between 1989 and 2004, combined with Irish HCV notification data for 2004–2009 [17]. After applying a number of assumptions in relation to reporting bias, under-diagnoses, establishment of chronic infection, and case fatality, the authors estimated a population prevalence of chronic HCV infection of between 0.5 and 1.2% in 2011.

Our findings for women aged 18–49 years are also in line with estimates from HCV antibody prevalence studies conducted in two Dublin hospitals on antenatal women in 2007 [16] and 2007–2008 [15], which estimated seroprevalences of 0.7% with 57% HCV RNA positive [16], and 0.9% with HCV RNA positivity of 64% [15], respectively. The slightly lower weighted seroprevalence among women aged between 18 and 49 years in our study is perhaps not surprising given that it represents a wider geographical area, with the earlier published studies having been performed in settings largely serving women from the greater Dublin area.

HCV is a notifiable disease in Ireland both by clinicians and laboratories. Our findings are also consistent with recent Irish HCV notification data in terms of age, sex and geographical area; 69% of Irish HCV notifications in 2014 were reported from HSE-East, with injecting drug use reported as the most common risk factor at 80% [18]. It seems plausible given the age-sex-geographical distribution of our data, that our findings could also be substantially influenced by the occurrence of HCV infection in people who currently inject drugs, or have done so in the past.

Since the introduction of screening of donated blood for HCV in the early 1990s, HCV transmission through blood and blood products is rare. Prior to that, however, around 1,700 cases of HCV infection were acquired through blood and blood products in Ireland; their disease history is being documented in the National Hepatitis C Database [19,20]. Some of these have been successfully treated with antiviral therapy; however, the group remains an important sub-group of the seropositive population in Ireland, and may explain some of the seropositive specimens we identified in older adults.

In a review of the natural history of HCV infection, Seeff and colleagues described chronic infection rates of up to 80% in studies of HCV-infected adults, with lower rates of ca 50% in infected children or young women [1]. Our overall estimated chronicity rate of 62% is at the lower end of this range, but the study was not designed to reflect the natural history of HCV infection in Ireland or to take into account factors such as antiviral treatment.

Compared with previous studies in Ireland, based on high-risk, localised or antenatal populations [11-16], this study has the advantage of being a national survey representative of the general adult population. Large in size, it provides good precision in our overall estimate and in selected subgroups. As it used specimens already collected for other diagnostic and screening investigations, it was relatively inexpensive to perform, and provided a population estimate in a short time frame.

The main limitation of our study is potential bias because individuals whose specimens are submitted to NVRL for testing are not likely to be completely representative of the general adult population. To minimise bias by age group and sex, we stratified the sampling frame before sampling, and sampled with probability proportional to the size of the strata in the general population. In addition, to adjust for geographical bias in sample selection and for under-sampling in three age-sex strata, we weighted for HSE area, age group and sex in the analysis.

Attempts were made to minimise potential bias by excluding certain categories of residual specimens from persons who would be expected to have a higher risk of being HCV-seropositive (e.g. specimens from STI clinics, drug treatment services, or those that were submitted specifically for HCV testing). The intent was to avoid the over-representation of persons in risk groups that might arise from inclusion of specimens from these sources. Due to the large number of specimens excluded on such grounds however, it could be argued that we have selected a sample biased towards low-risk specimens, and therefore the estimate obtained should be considered the minimum.

Compared with other study designs [15,21,22], we had limited opportunity to look at risk factors other than age, sex and geographical area, as the sample was drawn from residual sera. The anonymous nature of the survey also precludes us from knowing what proportion of these individuals are already aware of their HCV status, what proportion of resolved infections were consequent to antiviral treatment, and also prevents referral of patients with positive specimens to care pathways.

Notwithstanding these limitations, these data are the best estimates to date in Ireland of HCV seroprevalence in the general population and we believe they will serve to: (i) provide more accurate information for the public on their likely risk of infection; (ii) inform health service planning regarding future screening programmes, future burden of HCV-associated disease and demand for antiviral treatment in Ireland; and (iii) provide a benchmark for evaluating the effectiveness of primary and secondary HCV prevention programmes. Owing to its simplicity, low cost, and rapidity, we would also recommend this study design as a model for sero-epidemiological studies for other diseases in Ireland, or for HCV sero-epidemiological studies elsewhere in Europe.
